# Contamination of equipment in emergency settings: An exploratory study with a targeted automated intervention

**DOI:** 10.1186/1750-1164-3-8

**Published:** 2009-07-30

**Authors:** Chidi Obasi, Allison Agwu, Wale Akinpelu, Roger Hammons, Clyde Clark, Ralph Etienne-cummings, Peter Hill, Richard Rothman, Stella Babalola, Tracy Ross, Karen Carroll, Bolanle Asiyanbola

**Affiliations:** 1 Division of Surgery, Johns Hopkins Medical Institutions, Baltimore, Maryland, USA; 2 Division of Infectious Diseases, Johns Hopkins Medical Institutions, Baltimore, Maryland, USA; 3 The Applied Physics Laboratory, Johns Hopkins University, Baltimore, Maryland, USA; 4 Department of Computer Engineering, Whiting School of Engineering, Johns Hopkins University, USA; 5 Division of Emergency Medicine, Johns Hopkins Medical Institutions, Baltimore, Maryland, USA; 6 The Johns Hopkins Bloomberg School of Public Health, Baltimore, Maryland, USA

## Abstract

**Background:**

Despite standard manual decontamination, hospital equipment remains contaminated with microorganisms, contributing to nosocomial transmission and hospital acquired infections. This has the potential to negate the effects of healthcare workers' hand-washing protocols. In order to decrease the likihood of equipment contamination, there has been a rise in the use of disposable pieces of equipment, especially non-critical disposables. However, these carry a significant cost, both a direct financial cost (running into billions of dollars), as well as a cost to the environment. This is important because we hope to contain the cost of healthcare, one way to do that, is to look to the hospitals themselves, for innovative solutions that maintain the standard of care.

**Objective:**

To develop and evaluate the effectiveness of an simple decontamination device for use with portable hospital equipment, by comparing rates of residual contamination after use of the novel device versus those seen with standard manual decontamination methods.

**Methods:**

The Self-cleaning Unit for the Decontamination of Small instruments (SUDS) is a user-friendly, automated instrument developed via multi-disciplinary collaboration for decontamination in the clinical area. Pre- and post- utilization of portable medical equipment in an emergency department (ED) setting were cultured. To evaluate durability of the decrease in antimicrobial contamination, objects were re-cultured 48 hours after SUDS cleaning and following re-introduction into the clinical setting.

**Results:**

After manual decontamination, 25% (23/91) of the tested objects in the ED were found to be culture positive with clinically significant microorganisms(CSO). Fifteen percent (ED) of non-critical equipment tested had multiple organisms. Following the use of SUDS, the colonization rate decreased to 0%. Following SUDS treatment and re-introduction into the clinical settings, after 48 hours the contamination rates as reflected by the cultures remained 0%.

**Conclusion:**

Standard non-critical equipment is contaminated with clinically significant microorganisms. The SUDS device allows for effective and durable decontamination of hospital equipment of varying sizes in the clinical area without disrupting patient care.

## Introduction

The use of disposable non-critical items has increased over the years. This practice has been driven in large part by the known risk of fomite infection transmission and a lack of reliable alternatives to standard manual decontamination. A typical disposable item is used once and then discarded, resulting in hundreds of millions of dollars in annual costs. A midsized hospital, for example, may utilize over 30,000 units of disposable pulse oximeter sensors, at $9–$15 per unit [1]. Similar costs incurred across the US for this item only would yield millions of dollars per year (and larger costs for bigger hospitals), resulting in significant annual costs for this single item, disposable pulse oximeter sensors, running into hundreds of millions of dollars per year. Multiply that by the cost of all non-critical disposables and the costs will tend towards the astronomical. Until the cost-effectiveness of the various disposable items has been established, explicit attention should be paid to the cleaning and disinfection of equipment between patients [2].

According to Spaulding, noncritical equipment is defined as those items that come into contact with intact skin but not with mucous membranes [3]. Since intact skin is an effective barrier to most microorganisms, items that contact only the skin do not need to be sterile and may be cleaned where they are used [4]. In 1991, Favero and Bond provided a useful expansion of the Spaulding scheme by dividing the non-critical environmental surfaces into housekeeping surfaces and medical equipment surfaces [5].

Non-critical equipment used in the medical environment can, however, serve as fomites harboring microorganisms which can be transmitted [6-10] and contribute to nosocomial infections and hospital outbreaks [7,8]. Microorganisms such as Methicillin-resistant *Staphylococcus aureus *(MRSA), Vancomycin-resistant enterococcus (VRE), and gram negatives can survive on inanimate objects such as hospital equipment for many months [8,9]. MRSA is now endemic and even epidemic in many U.S. hospitals and long-term care facilities and is of particular importance to ambulatory and ED settings in many communities [11-15]. When clean (washed) hands or gloved hands touch contaminated objects, they become contaminated with similar organisms, which can then potentially be transmitted to other surfaces and people [16,17]. Inadequately disinfected reusable equipment has been reported to be a source of nosocomial infection, particularly with regard to MRSA and VRE. Nosocomial infections [NI], reportedly account for up to 80,000 U.S. deaths each year, according to the Center for Disease Control and Prevention (CDC) [18,19]. Accordingly, the CDC and the Healthcare Infection Control Practices Advisory Committee (HICPAC) recommend routine decontamination of all hospital equipment [20].

Manual decontamination is often unsatisfactory due either to ineffectiveness of decontamination protocols or to poor compliance with these protocols, with commonly used equipment in the hospital frequently remaining contaminated after manual decontamination [9]. Particularly problematic have been multi-planar/configurational devices that challenge traditional cleaning modalities. There has been scant attention dedicated to high-volume areas with rapid patient turnover such as the ED (and other ambulatory settings) compared to comparatively low-turnover inpatient settings, e.g., long-term care and intensive care units where most studies to date have been conducted [7,10-12,17,21,22]. Until now, there has not been a method of automated multimodal and multi-configurational image distance-based decontamination of hospital equipment in the clinical area that does not interfere with patient flow. An ideal device would allow for safe, effective, and efficient decontamination of portable hospital equipment of varying sizes in the clinical area. Such a device would be useful in high-volume clinical areas. Looking to the precedent set by operating rooms, exemplified by their ability to effectively sterilize instruments for reuse due to a process- and device-based standard of care, the self-cleaning unit for the decontamination of small objects (SUDS) was developed.

The objective of this study was to develop a device to more effectively decontaminate portable hospital equipment in comparison with standard manual decontamination and to evaluate differences in bacterial contamination rates. Our specific hypothesis is that an automated unit for the decontamination of medical equipment provides a safe, effective, means of decontaminating hospital equipment of various sizes and configurations in high-volume clinical areas without interfering with patient flow.

## Methods

### Overview

**Figure 1:**
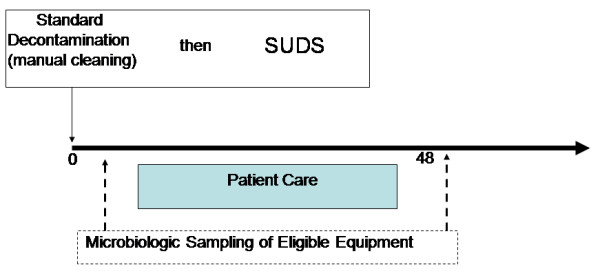
**Schematic of Procedure in Emergency Department**.

Prospective comparative study of the effectiveness of standard manual and SUDS decontamination of the patient care area in the ED. In brief, results of microbiologic sampling of medical and electronic equipment in patient care areas conducted following standard manual decontamination was compared to microbiologic sampling following use of the SUDS. In this setting, staff members were blinded as to the timing of the proposed research. In the ED setting, instruments were placed in the SUDS immediately after manual decontamination and replaced in the patient care area/bed space for use on subsequent patients (see figure 1).

### Setting

The adult ED of Johns Hopkins Hospital (JHH) sees over 58,000 patients annually. The ED has 32 bed spaces, excluding acute specialty care spaces. There are 14 patient care areas, which for the purposes of this study, were defined as enclosed areas that may contain one or more beds having one entrance and exit. Inclusion criteria: all portable equipment (i.e. any detachable, movable, and reusable equipment) as well as all electronic devices utilized in association with patient care areas and bed spaces (e.g., phones and keyboards). Exclusion criteria: Non-detachable and non-movable equipment (e.g. light fixtures). In addition, bed spaces outside the patient care areas, bed spaces in specialty areas such as acute psychiatric care areas, and environmental surfaces were not included in this study. Electronics in shared spaces were excluded, as manual decontamination could not be assured during turnover of individual patients. ED Procedures: The ED component of the study was carried out over a four-day period. Standard practice in the ED is manual decontamination of the clinical area and equipment in the patient care area following each patient discharge and prior to bringing a new patient into the patient care area. Therefore, swabbing is done during turnover, just prior to the admission of a new patient into the patient care area or bed space.

### Equipment

Equipment was defined as portable if it was detachable, movable, and reusable with patient contact. Electronic equipment in clinical areas was also included in this study as a separate category. In the ED, equipment was classified into three predefined groups: 1) stands, 2) cables, and 3) electronics. For the purposes of this study, portable electronics included either keyboards or phones. Most patient care areas had one keyboard and phone each, both of which were swabbed. In areas where the electronics were a shared resource (e.g., one phone and keyboard serving multiple patient areas), the shared electronic equipment was excluded. Stands included all wheeled, portable equipment (e.g., intravenous poles, sphygnometers). All stands present in the patient care areas during the study period were swabbed. Cables included all portable equipment that was in cable form such as EKG leads and cables, blood pressure cables, pulse oximeter cables, etc., that were used for contact with the patient. Using a random sequence generator, one cable was chosen randomly in each patient care area for sampling.

Sampling for microbiologic assessment: For all devices/equipment, the site of culture was predetermined and standardized prior to initial swabbing. Therefore, the same site was chosen for swabbing after both manual and automated decontamination. A 3 cm^2 ^area was marked and swabbed on each instrument. Samples collected were semi-quantitatively assayed for microorganisms. A single investigator swabbed the equipment (see table 1).

**Table 1:** Equipment included in this study [Emergency Setting]

**Stands**	
Intravenous poles	6
Ultrasound machines	3
Blood pressure stands	9
Pulse oximeter stands	6
Mayo stands	3
Blood pressure monitor	3
Miscellaneous	8

**Electronics**	

Wall phones	7
Keyboards	14

**Cables**	

Blood pressure	16
Pulse oximeter	8
Ekg cables	8

### Intervention

The self-cleaning unit for the decontamination of small instruments (SUDS) is a multimodal portable decontamination unit. This unit allows for primary, secondary, and tertiary decontamination mechanisms with aerosolized biocide, ultraviolet light, and dry heat, respectively. Surface and base rotation via a clockwise and counterclockwise mechanism serves to increase the exposure of equipment to the biocide by optimizing air flow directionality. Turbulence generated at the base allows for air flow patterns that increase exposure to the undersurface of the equipment. Image distance-based techniques allow for maximum intervention in specific areas. S-shaped curvatures at the edges of the surface rotatory mechanism allow for the attachment of multiple devices to the surface rotatory mechanism. Air cleaning and filtration modes allow for the expulsion of clean air into the environment; this may be connected to the facility filtration system. Only the primary mode of decontamination (aerosolized biocide) was used in this study.

The advantages of the SUDS are consistency in automated dispensation and the design of rotational and turbulence forces to optimize dispersal. In addition, targeted image distance-based intervention to specific areas allows for maximal exposure of devices of all configurations. Variability in the choice of biocide and the multimodal approach limits the likelihood of the development of biocide resistance. Adjuncts include mechanical, humidity, and pressure-based interventions. Air cleaning allows for use in the clinical area without interfering with patient flow. Cycle time is variable depending upon the biocide used, the targeted and standard dispersal of same, and the ultraviolet light. During this study, cycle time was 30 m for up to 15 instruments of varying configurations in one cycle. The SUDS was derived from the operating room model of instrument care where instruments are sterilized locally and reused in an automated fashion within a culture of staff maintained cleanliness. Since non-critical medical equipment does not need to be sterilized but merely decontaminated, this decontamination can be done on site [23].

### Biocide

For the purposes of this study, a commonly available biocide, Sporicidin^®^, was used. Sporicidin^® ^is a 1.56% phenol and 0.06% sodium phenate solution that can be delivered in aerosolized form. Sporicidin^® ^Disinfectant Solution is FDA 510(k)-cleared, EPA-registered for hospital use, and compliant with OSHA Blood-borne Pathogens Standards (29 CFR 1910.1030) [24]. It is used for both direct manual decontamination and aerosolized delivery per manufacturer's directions. SUDS allows for the use of most biocides that can be delivered in an aerosolized form, allowing for versatility of type, concentration, and duration of biocide delivery, which minimizes microbial resistance. Manual cleaning was carried out with AIREX 109 per usual hospital protocol. This is a quaternary ammonium compound. We were unable to get aerosolized versions.

### Microbiologic Analysis

Rayon swabs (Copan Diagnostics, Corona, CA) were used to obtain cultures. Swab cultures were plated onto trypticase soy agar with 5% sheep blood (SBA) and MacConkey agar, and the swab was then put into a Schaedler's broth to incubate overnight. The SBA and MacConkey plates were plated semi-quantitatively. The broth was included to enrich organisms present in small quantities. If the broth was turbid after 24 hrs of incubation, it was sub-cultured to selective media to evaluate for the presence of methicillin-resistant *Staphylococcus aureus *(MRSA), vancomycin-resistant Enterococci (VRE), and resistant gram-negative organisms. From the selective media, only potential MRSA, VRE, and resistant gram-negative organisms were completely characterized. All cultures were incubated for 48 h at 35°C under aerobic conditions. Contamination was defined as equipment containing more than 5 colony-forming units (CFUs)/cm^2^. Clinically significant organisms were defined as those known to cause human disease in a nosocomial setting (MRSA, VRE, resistant gram-negative rods, and fungi).

### Statistical Analysis

The total population of portable items present within a shift that met criteria for this study was defined. This included two cables per bed space, all electronics in the patient care area, and all stands present during the shift. With a 95% confidence interval and 4.5% confidence level, a sample size of 91 instruments was obtained. One cable from each bed space was randomly selected from the minimum 2 cables present using a random sequence generator, while convenience sampling of the stands and electronics was carried out. All electronics as defined in this study from each patient care area and all stands present during the study period were included.

## Results

**Figure 2:**
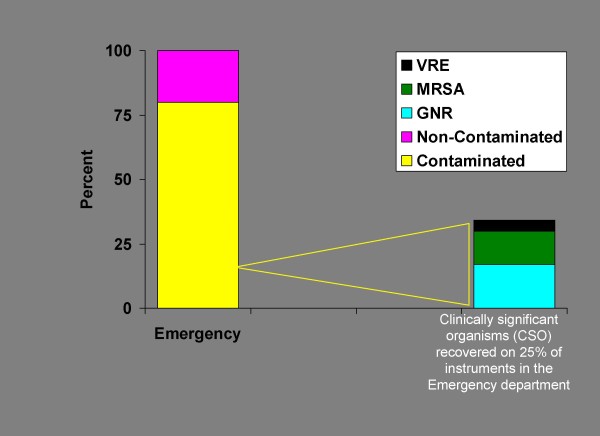
**Overall Contamination Levels in the Emergency Department**.

Baseline level of contamination were measured following standard ED manual decontamination procedures. A total of 182 swabs were taken from predetermined areas on 91 pieces of selected equipment from bed spaces and patient care areas. The various instruments sampled are detailed in Table 1; however, in brief, 14 keyboards, seven wall phones, all 38 stands associated with patient care areas during a shift, and at least one cable from all bed spaces (32) were swabbed. Eighty percent of instruments were contaminated (Figure 2), and 25% (22/91) were contaminated by clinically significant organisms (CSO). It is notable that 75% of equipment was lightly or moderately contaminated (< 50 CFUs/cm^2^) following manual decontamination; the remaining equipment was > 50 CFUs/cm^2^. All heavily contaminated equipment had positive cultures.

**Figure 3:**
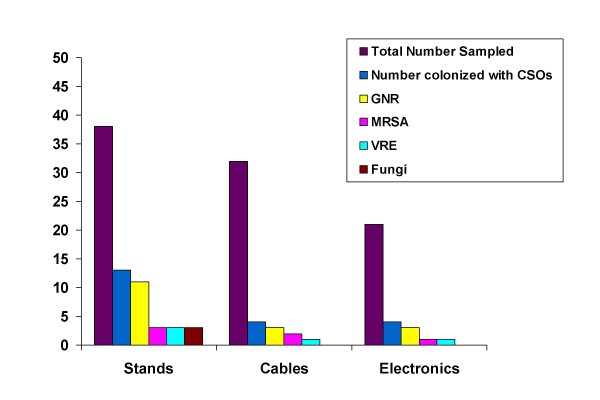
**Instrument Contamination by Type of Organism: Emergency Department**. Note: instruments may be colonized with > 1 clinically significant organism (CSO).

The most common recovered clinically significant organisms were the gram-negative rods, which were found in 16.5% (15/91) of instruments. Twelve percent (11/91) of instruments had MRSA, 4.4% (4/91) had VRE and fungi, respectively. Fourteen percent (13/91) had more than one organism, and 5.5% (5/91) of instruments had more than 3 organisms. Also, 8.8% (8/91) of instruments had more than one type of GNR. The most common equipment type contaminated with clinically significant organisms was the stands (13/38) 34.2%, while 19% (4/21) of electronics, and 12.5% (4/32) of cables were contaminated (see Figure 3). Of the 13 contaminated stands, 23%(3/13) were contaminated with VRE, 23% (3/13) with MRSA, 84.6%(11/13) with GNRs and 23% (3/13)) with fungi. Fifty-four percent (7/13) contaminated stands had more than one organism (Figure 3).

For the intervention phase, all instruments were placed in the SUDS for 20 minutes of decontamination and 10 minutes of "stand time." There was no growth of CSOs following SUDS decontamination (p < 0.001). Forty-eight hours after the initial automated decontamination, all previously contaminated emergency room equipment was re-swabbed in the same previously defined area. All of the previously contaminated equipment was found to be free of CSOs 48 hours later. All electronic equipment remained fully functional following SUDS decontamination.

## Discussion

Based upon the risk of potential transmission of hospital acquired (nosocomial) pathogens, the CDC and multiple leaders in the field [8,9,20] have suggested that routine cleaning of medical equipment with antimicrobial agents, especially in the immediate vicinity of patients, is recommended. Maintaining the chain of cleanliness, decreasing microorganism load, and targeted eradication of sources of infection have already been shown to decrease hospital acquired infections and eliminate outbreaks [[8,9], and [23]]. Clinically significant organisms have been shown to persist on equipment, sometimes for months [23,25]. A review of the mostly inpatient setting literature reveals that non-critical hospital equipment as well as electronics such as keyboards and wall phones harbor pathogenic and non-pathogenic bacteria. Rates of contamination of up to 100% with varying pathogenic organisms have been described [9]. Boyce et al. reported a contamination rate of 25% with MRSA for an intravenous pole in the inpatient setting (although this was in the vicinity of colonized patients), while Rutela described rates of up to 36% for GNR and 12% VRE on keyboards [10,26,27]. Even when hand hygiene is strictly maintained before and after contact with a patient, washing hands after each piece of medical or electronic equipment is touched would impede patient care to a degree of impossibility. Various researchers have found that hands (even gloved hands) with which one might touch instruments can immediately become contaminated with VRE in a substantial proportion of cases [16,17]. Poor hand hygiene compliance can only potentiate this possibility [28].

The significant levels of contamination with clinically significant organisms in our study (25% of instruments in the emergency room) and others suggest that the possibility of colonization of other personnel, subsequent patients, and other equipment through the hands of healthcare workers and patients is of concern, given the propensity of clean hands to become re-colonized after contact with a contaminated piece of equipment while caring for a particular patient. Given the varied nature of the population that visits emergency departments and clinics, including immune-suppressed patients and patients without intact skin barriers, transmission of such organisms from contaminated equipment ideally should be avoided. Various (and virtually any) organisms can cause infection if they come into contact with a susceptible patient [29] and this is more likely in the emergency or ambulatory setting. The SUDS appears to provide a mechanism for eliminating equipment contamination, thereby preventing non-critical equipment from serving as an incubator and transmitter of clinically significant microorganisms.

There is scant literature on the incidence/prevalence of equipment contamination in areas where community and hospital mix in a rapid and high-turnover situation with a wide variety of patients of varying clinical complexity and needs that then disperse to a large number of different destinations, as represented by the ED in our study. Most studies to date on contamination rates of hospital equipment have been conducted in the inpatient setting [7,8,11-13,22,24], particularly the intensive care unit (ICU). However, the patient turnover in a single ED bed space (with its associated cables, stands, and electronics) compared to a single ICU bed is over 20 to 1 per year and 10 to 1 in the surgery clinic compared to an ICU bed (based on annual volumes for 2006). Studies evaluating the use of chemical and ultraviolet decontamination of equipment and environmental surfaces have required emptying and sealing off the room during the decontamination process, which is currently prohibitive in high-turnover clinical areas [30-32]. To our knowledge, this is the first study to evaluate the incidence of contamination of hospital equipment and electronics and a targeted automated intervention for same in this high-volume, high-turnover clinical setting without interruptions to patient thoroughfare.

When done in a meticulous fashion, manual decontamination has been shown to significantly decrease colonization rates in parts of certain instruments [9]; however, there are practical limitations to efficient manual decontamination in day-to-day practice. Hospital equipment is of variable configuration, leading to difficulty with manual decontamination. Adequately training personnel to clean specific devices optimally is resource-intensive. Further, some equipment is heavy or awkward; for example, many stands have a center of gravity that predisposes them to tipping over easily, increasing the propensity for accidents involving staff and, potentially, patients when equipment is tipped to the side in order to thoroughly decontaminate it. With the increasing number of patients cared for in ambulatory settings and the high turnover rate, efficient and effective manual decontamination is simply time-challenged. In a global, systemic review of all published and unpublished studies evaluating levels of contamination of healthcare equipment spanning a 32-year period, Schabrun et al. found up to 17 different organisms on a range of medical equipment [9]. This suggests that current decontamination protocols are ineffective or not adhered to by healthcare professionals. The current trend is to utilize more and more non critical disposables. Given all the above, it is clear that alternative approaches to this issue are warranted unless we expect that all hospital equipment, no matter how complex, will become disposable in the future, accepting the exponential costs both in cash and kind (environment).

**Figure 4:**
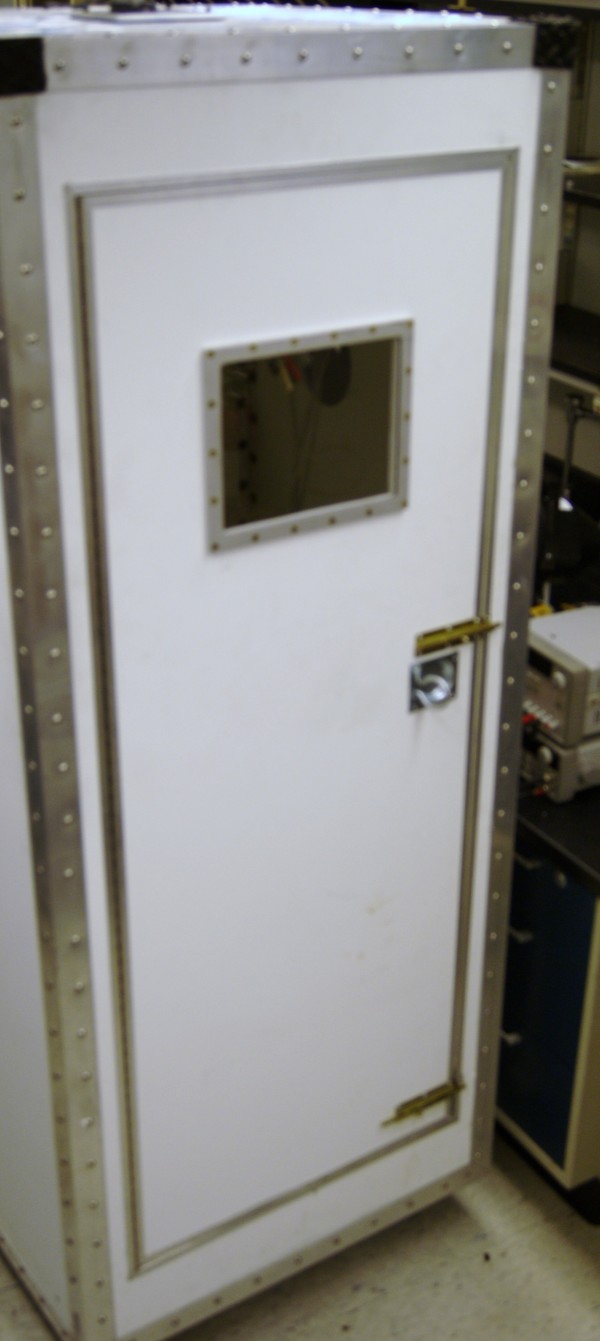
**Photo of the Self-cleaning Unit for the Decontamination of Small instruments**.

**Figure 5:**
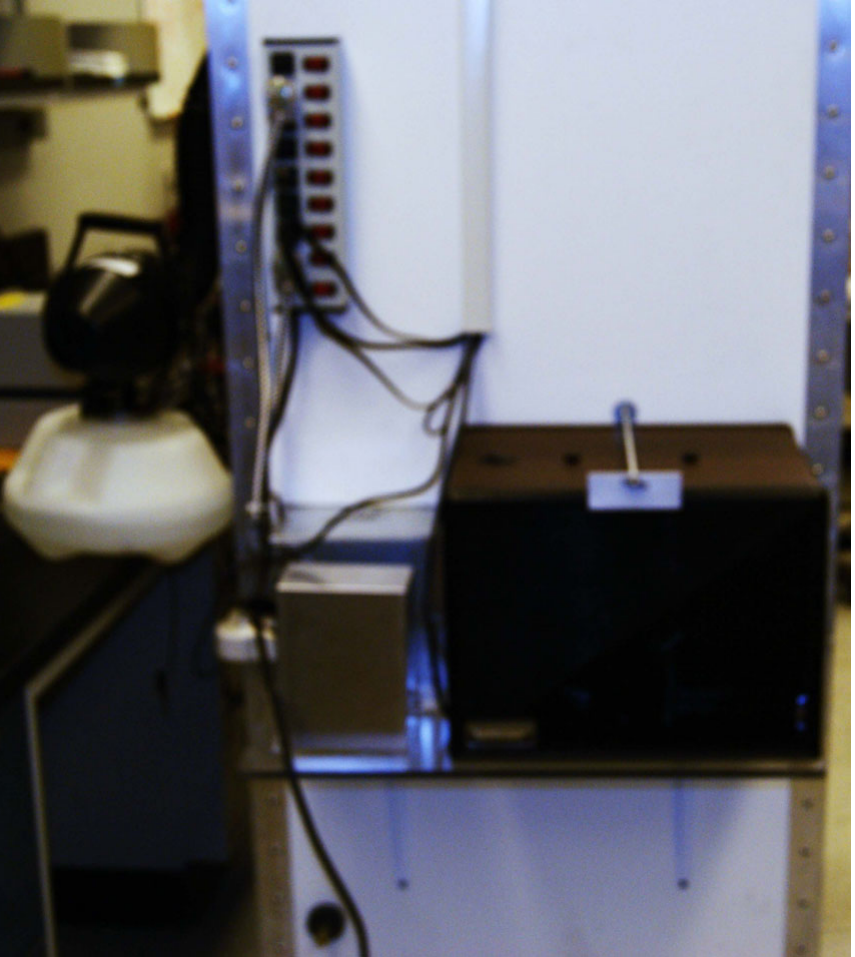
**Photo of the Self-cleaning Unit for the Decontamination of Small instruments**.

The SUDS (figures 4 and 5) provides a multimodal self-cleaning means of decontaminating a wide variety of equipment for staff in high-volume, high-turnover clinical settings. In this study, we showed that the SUDS eliminated clinically significant organisms after a single cycle. The manpower needs were minimal, and the equipment was shown to be free of clinically significant organisms 48 hours later, even after routine use in the emergency department. Automating the process of decontamination of most equipment could potentially allow increased attention to areas closest to patients, such as bedrails, that are likely to be contaminated but are not amenable to detachment and automated decontamination. This in turn could enhance overall compliance and culture change. Automation allows for the maintenance of consistency in the decontamination process, in addition to more effective and targeted dispersal of biocide via rotational, turbulence, and image-based means. Obviously, bacteria cannot be totally eliminated from the environment but equipment in this preliminary study showed that they were free from CSO by culture 48 hours later, having been put through normal use in the emergency room in the 48 hours since commencement of the study.

## Limitations

We postulate that the main advantage of the SUDS is the dispersal of the biocide over the entire piece of equipment, irrespective of its type. However, further, more robust studies will need to be carried out to define this further, in view of the differences in the nature of the biocides utilized in this study.

This study assessed the incidence of colonization of portable and electronic equipment as a group. Further studies would be needed to assess the precise nature and incidence of colonization of individual pieces of equipment within the same ambulatory or emergency setting. The study only assessed re-colonization rates 48 hours after SUDS and therefore cannot comment on resistance of devices to re-colonization beyond 48 hours. Additionally, the 48-hour time frame would be dependent upon the combination and duration of levels of decontamination utilized in a particular cycle. We did not specifically quantify the difference/impact of patient flow between manual decontamination and utilization of the SUDS in this study; however, patient care continued without interruption during the run of this trial in the emergency setting. Only patient care areas and bed spaces were evaluated, and this may lead to an imprecise estimation of the incidence of colonization. Equipment in the central stations in the emergency room was not included in this study, also possibly leading to underestimation. As this study primarily was aimed at evaluating elimination of equipment contamination, neither contamination of environmental surfaces nor levels of hand hygiene were evaluated as part of its scope. However, the tested equipment in this study remained free of clinically significant organisms after 48 hours of subsequent use in a busy emergency area, regardless of the levels of other confounders such as hand hygiene and environmental contamination.

There are multiple other issues that require further investigation. The precise incidence of infection of patients after contact with contaminated equipment or the colonized hands of healthcare workers is not precisely known. The degree to which the environment (which includes hospital equipment in some studies) is implicated in the direct transmission of organisms resulting in NI (nosocomial infections) has been debated. Hand hygiene compliance and surface environmental decontamination have been confounding variables affecting precise quantification in many studies and are worthy of further study. However, standard of care currently recommends decontamination of non-critical equipment.

The impact of automated decontamination on overall cost compared with that of disposable equipment should be the subject of future analyses. Frequently in the case of disposable pulse oximeters, the quality is cited as a reason for it to be made as a disposable item. The quality is a distant secondary concern because we can make better reusable equipment: unfortunately, there is no incentive to do so, as detailed by Stewart almost 13 years ago [33]. If millions of dollars in a single hospital are spent on, for example, disposable pulse oximeter sensors, we should be able to quantify the number and types of infections disposable pulse oximeter sensors have prevented. And if we don't know the answer to those questions, shouldn't we be asking ourselves: why not?

## Summary

There is scant data evaluating contamination of non-critical equipment in the emergency room setting, which differ from the inpatient setting in volume, turnover rate, and variety of clinical conditions seen. Contamination of equipment has been reported in various inpatient settings, and transmission to multiple patients is a possibility even with strict hand hygiene before and after individual patient contact, as evidenced by nosocomial outbreaks linked to contaminated equipment. Our study showed that, with current manual decontamination methods, significant levels of contamination were identified, serving as a potential vector for transfer of organisms to personnel and subsequent patients. The SUDS provides an effective, efficient, and user-friendly multimodal method for decontaminating a wide range of hospital equipment in the clinical area in an automated fashion that is far superior to the current standard of manual decontamination. It can, therefore, potentially be an effective mechanism for decontamination of non-critical disposables and may provide an alternative to the use of disposables, particularly as the SUDS can decontaminate a wide variety multi-modal and multi-configurational equipment.

## Competing interests

The authors declare that they have no competing interests.

## Authors' contributions

BA conceived of the study, designed and carried out the study, as well as prepared and edited the manuscript. CO performed the cultures and data collection and assisted in drafting the manuscript. TKC contributed to the design of the study, assisted with drafting the manuscript, edited the manuscript and assisted with microbiologic analysis. AA contributed to the design of the study, edited the manuscript, and assisted with microbiologic analysis. TR carried out the microbiologic analysis. BA, WA, RH, REC, and CC all contributed to the engineering works on the device. PH and RR contributed to the design of the study, and edited the manuscript.

BS contributed to the design of the study and carried out the statistical analysis. All authors read the final manuscript.
